# Unencapsulated Air-stable Organic Field Effect Transistor by All Solution Processes for Low Power Vapor Sensing

**DOI:** 10.1038/srep20671

**Published:** 2016-02-10

**Authors:** Linrun Feng, Wei Tang, Jiaqing Zhao, Ruozhang Yang, Wei Hu, Qiaofeng Li, Ruolin Wang, Xiaojun Guo

**Affiliations:** 1 National Engineering Laboratory of TFT-LCD Materials and Technologies, Department of Electronic Engineering, Shanghai Jiao Tong University, Shanghai 200240, China

## Abstract

With its excellent mechanical flexibility, low-cost and low-temperature processing, the solution processed organic field-effect transistor (OFET) is a promising platform technology for developing ubiquitous sensor applications in digital health, environment monitoring and Internet of Things. However, a contradiction between achieving low voltage operation and having stable performance severely hinder the technology to become commercially viable. This work shows that, by reducing the sub-gap density of states (DOS) at the channel for low operation voltage and using a proper low-*k* non-polar polymer dielectric layer, such an issue can be addressed. Stable electrical properties after either being placed for weeks or continuously prolonged bias stressing for hours in ambient air are achieved for all solution processed unencapsulated OFETs with the channel being exposed to the ambient air for analyte detection. The fabricated device presents a steep subthreshold swing less than 100 mV/decade, and an ON/OFF ratio of 10^6^ at a voltage swing of 3 V. The low voltage and stable operation allows the sensor made of the OFET to be incorporated into a battery-powered electronic system for continuously reliable sensing of ammonia vapor in ambient air with very small power consumption of about 50 nW.

Organic field-effect transistors (OFETs) have received worldly wide research attention, owning to their attractive features of sustainable performance improvement and functionalization through chemical structure tailoring, superior intrinsic mechanical flexibility, low temperature and fast processing, and compatibility with arbitrary substrates (plastic, paper and fabric)^1^,^2^,^3^,^4^,^5^,^6^. These features of OFETs ideally match well to ubiquitous sensors, which are demanded as part of global issue solutions for digital health, environment monitoring and Internet of Things^7^,^8^,^9^. The sensors, using OFETs as transducers, could be developed with low-cost and great freedom for sensing function integration on arbitrary substrates to work with multi-physics or bio/chemistry signals^10^,^11^. As opposed to other implementations such as a chemiresistor, OFET based sensors can provide better sensitivity through the amplification of the transistors^12^,^13^,^14^,^15^. However, most of these targeted sensor applications are portable or wearable, requiring the OFETs to be integrated in severely power-constraint electronic systems with battery or a. c. field. Good enough operational stability under electrical bias storage and lifetime are also prerequisites for practical use.

In the last decades, remarkable progresses have been made on development of high mobility soluble organic semiconductors^16^,^17^, and solution or printing based fabrication techniques to develop a commercially competitive manufacturing approach for OFETs^18^,^19^,^20^. However, the demonstrated large operation voltage of typically a few tens volts, operational and storage instabilities in ambient air remain to be the main bottlenecks preventing further advances towards the targeted applications. Intensive studies have thus been made on improving the device stabilities^19^,^20^,^21^ and reducing the operation voltage^22^,^23^ for OFETs, respectively.

For OFETs in encapsulated or operated in inert environment, the bias stress effect (BSE) induced threshold voltage shift under continuous bias during operation is the most studied issue^24^. The main mechanism is thought to be the trapping of carriers from the gate bias-induced conduction channel into less mobile localized states^25^. The trapping probability is proportional to the local trap states, the gate induced electrical field strength and the carrier density in the channel. Therefore, to reduce the BSE, it will be important to form a high quality semiconductor/dielectric interface. Weak gate electrical field and low carrier density during operation is also helpful. For OFETs in an unencapsulated structure, the channel layer is exposed to the ambient air. As a result, besides BSE, oxidative degradation of the semiconductor layer, and absorption of polar H_2_O molecules on the semiconductor and the dielectric films can also happen to cause device degradation^26^,^27^,^28^. Therefore, for unencapsulated OFETs, to achieve the required long-term stability during storage and operation, air stable and water repellent semiconductor and dielectric materials are more urged. In the past, several air stable organic semiconductors have been developed by either modifying the side chains^29^ or removing/blocking the oxidatively susceptible sites in the molecules^30^. It was also concluded from experiments that less polar dielectric free of -OH groups can effectively suppress water adsorption for better device stability^31^,^32^.

On the other hand, to reduce the required operation voltage of OFETs, the widely used approach is to enlarge the gate dielectric capacitance with ultra-thin^33^ or high-*k*^34^ dielectric layer. However, a very thin dielectric layer is hard to be formed by simple printing or coating processes, and will cause large gate leakage current, and deteriorate the device functionality and reliability under electrical bias stress^35^. Moreover, a thin dielectric layer can also strengthen the gate electric field, and thus enhance charge trapping. For high-*k* gate dielectrics, the polar surface groups tend to trap carriers from gate bias induced conduction channel or interact with water from surrounding ambience if the devices are not well encapsulated^24^. The resulted localization of charge carriers or formation of Fröhlich polarons can cause not only mobility degradation^36^, but also increased hysteresis and device instabilities^37^. As a result, a contradiction between achieving low voltage operation, and having stable device performance severely hinder solution processed OFETs to become commercially viable for the targeted ubiquitous sensor applications.

According to the field-effect transistor (FET) theory^38^, the required voltage swing to switch a transistor is highly dependent on the subthreshold swing (*S*), which can be described as:


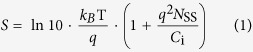


where *k*_B_ is the Boltzman’s constant, T is the absolute temperature, *N*_SS_ is the effective sub-gap DOS at the channel, q is the elementary charge, and *C*_i_ is the specific gate dielectric capacitance.

From equation 1, it can be seen that the required operation voltage for a FET depends on not only the gate dielectric capacitance, but also the effective sub-gap DOS at the channel. Therefore, if *N*_SS_ was effectively reduced, low voltage operation could also be realized. Our previous study reveals that, it is feasible to reduce the effective sub-gap DOS at the channel through blended solution of small molecule organic semiconductor and insulating polymer binder, and OFETs of steeper *S* can thus be realized for low voltage operation with less strict requirements on the capacitance of gate dielectric layer^39^,^40^,^41^,^42^. This work shows that, based on this material system to reduce the operation voltage, the contradiction between achieving low voltage operation and having stable device performance can be addressed for all solution processed OFETs by selecting a low-*k* non-polar polymer dielectric layer. The devices were implemented in a bottom-gate bottom-contact unencapsulated configuration, with the channel being exposed to the ambient air for analyte detection. Blend of small molecule acene semiconductor and insulating polymer binder was simply drop-casted on a thick and low-*k* poly(vinyl cinnamate) (PVC) gate dielectric layer to form a small sub-gap DOS channel without needing complex interface engineering processes. The source, drain and gate electrodes were formed by inkjet printed (IJP) silver.

The fabricated device presents a steep *S* less than 100 mV/decade, enabling an ON/OFF ratio of 10^6^ with a voltage swing of 3 V. Even without encapsulation, the devices show very stable electrical properties after either being placed for weeks or continuously prolonged bias stressing for hours in ambient air. The excellent features of low voltage and stable operation allow the sensor tag made of the OFET to be incorporated into a self-designed 3.7 V lithium-battery powered electronic system for long-term reliable sensing of ammonia vapor in ambient air. The power consumption of the OFET sensor tag is as low as 50 nW.

## Results

### Device structure and processes

**Figure 1:**
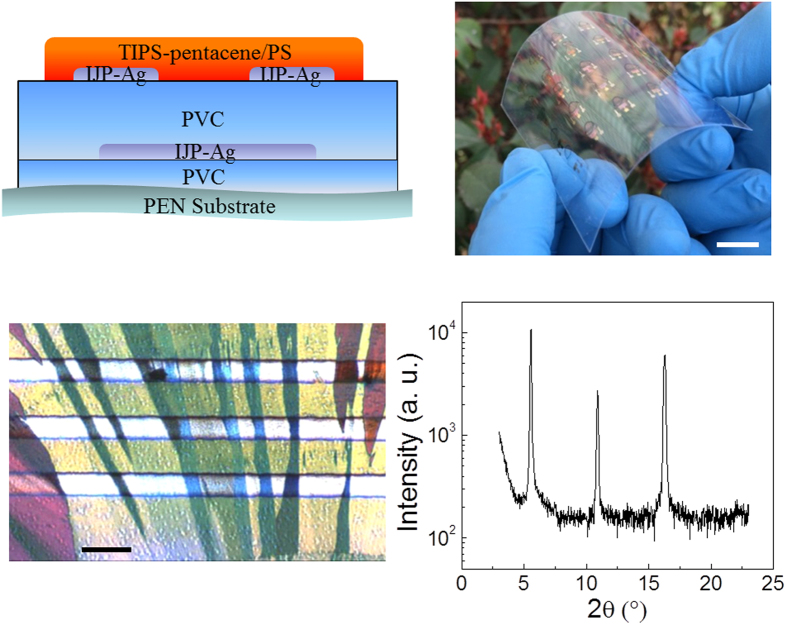
Device structure of the fabricated all solution processed OFETs. (**a**) The schematic diagram of the fabricated bottom gate bottom contact OFET devices. (**b**) The photo image of the 6 cm × 6 cm flexible sample. The scale bar is 1 cm. (**c**) The top-view polarized optical micrograph of the channel area for the device. The scale bar is 100 μm. (**d**) The X-ray diffraction (XRD) spectrum of the semiconductor for the device.

Figure 1(a) illustrates the bottom gate bottom contact (BGBC) device structure of the fabricated OFETs with the channel being exposed to the ambient air for analyte detection. The photo image of an array of OFETs fabricated on 6 cm × 6 cm size polyethylene naphthalate (PEN) substrate is given in Fig. 1(b). Silver gate, source and drain electrodes are formed by IJP of metal-organic precursor type ink, followed by a 15 min annealing process at 145 °C. A 295 nm thick non-polar PVC film (dielectric constant of 3.4) is used as the gate dielectric layer, resulting in a dielectric capacitance of 10.2 nF/cm^2^ at 20 Hz. The sample was then placed on an inclined support (a glass slide with the tilt angle of 10°), and blend of 6,13-bis(triisopropylsilylethynyl)-pentacene (TIPS-pentacene) and polystyrene (PS) in chlorobenzene is drop casted on the surface of the source-drain contacts and the PVC dielectric layer using a micropipette to form the semiconductor layer. The inclined structure help to form long crystalline TIPS-pentacene domains oriented along the channel^43^, as shown in Fig. 1(c). The X-ray diffraction (XRD) spectrum in Fig. 1(d) shows high diffraction peaks at 5.56°, 10.9° and 16.28°. The spectrum agrees well with previously reported results, indicating well-ordered triclinic structure of the formed semiconductor film^44^.

### Low voltage operation

**Figure 2:**
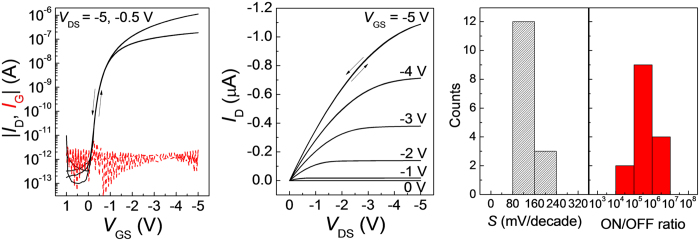
The measured representative electrical characteristics of the fabricated device with the channel length of 80 μm and channel width of 1600 μm. (**a**) Transfer characteristic (*I*_D_−*V*_GS_), (**b**) Output characteristic (*I*_D_–*V*_DS_) and (**c**) The histograms of extracted subthreshold swing (*S*) and ON/OFF current ratio for 15 devices over a 6 cm × 6 cm size substrate.

Figure 2(a,b) show the measured representative transfer and output characteristics of the devices. Despite the small specific gate dielectric capacitance of 10.2 nF/cm^2^ as shown in Fig. S1(a), the device presents a steep *S* as small as 97 mV per decade. The gate leakage current is favoritely low attributed to the relatively thick polymer dielectric layer. The extracted values of *S* and ON/OFF current ratio from the transfer characteristics for 15 devices over a 6 cm × 6 cm size substrate (Fig. S1(b)) are given in Fig. 2(c). The extracted mobility and threshold voltage (*V*_th_) values for the 15 devices are given in Fig. S1(c). The relatively thick (295 nm) gate dielectric layer help to achieve good yield for the low voltage devices even when all processes were completed in a non-cleanroom lab environment. The field-effect mobility in the saturation regime, *μ*_sat_, is extracted to be around 0.6 cm^2^/(V.s), using the expression *μ*_sat_ = 2*L*/*WC*_i_(∂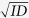/∂*V*_GS_)^2^ at a gate-source voltage of −3 V, where *I*_D_ is the drain current, *V*_GS_ is the gate-source voltage, *C*_i_ is the specific gate dielectric capacitance, and *L* and *W* are the channel length and width, respectively. As shown in Fig. S2, the mobility value is normal for TIPS-pentacene based devices, but is extracted at a much lower gate electrical field. The deep *S*, low gate leakage and high mobility enable to achieve an ON/OFF current ratio of 10^6^ with a voltage swing of 3 V. It can be seen from Table 1 that, in terms of the achieved ON/OFF ratio and the required voltage swing, the device in this work presents the best performance among all solution processed OFETs, which is even comparable with all vacuum processed low voltage OFETs with a nanometer scale thick inorganic gate dielectric layer^45^. The performance is attributed to much lower effective sub-gap DOS at the channel, which is of orders lower than previous work^34^,^45^,^46^,^47^,^48^,^49^,^50^, as compared in Table 1. The effective sub-gap DOS *N*_ss_ is extracted based on equation 1.

The exhibited negligible hysteresis in both output and transfer characteristics also indicates low trap interfaces formed between the semiconductor layer and the gate dielectric layer, and the semiconductor layer and the ambient air. These high quality interfaces are prerequisites for achieving stably operated OFETs.

### Operational and shelf lifetime stabilities

**Figure 3:**
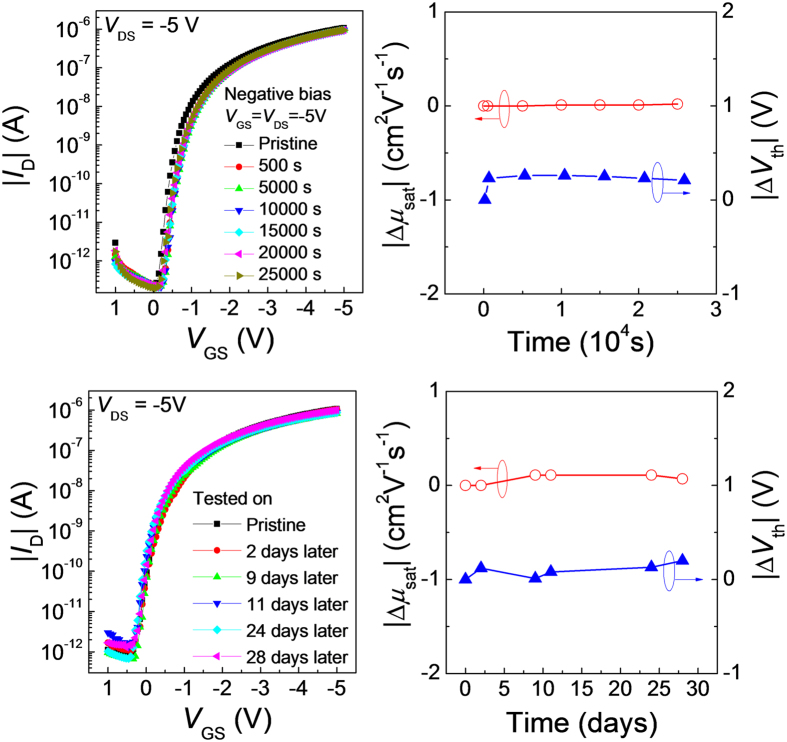
Operational and shelf lifetime stabilities of OFETs. (**a**) The measured bias stress stability of the device with an applied bias voltage of *V*_GS_ = *V*_DS_ = −5 V for 25000 s. (**b**) The relative changes of extracted mobility and threshold voltage of the devices as a function of bias time. (**c**) The measured transfer characteristics of the device during the four weeks’ storage in ambient air environment. (**d**) The relative changes of extracted mobility and threshold voltage of the devices as a function of time. The relative humidity and temperature at each measurement over the four week are given in Fig. S4(a).

For practical sensor applications, both operational and storage stabilities and under continuous electrical bias are required. The measured transfer electrical characteristics and the relative changes of extracted mobility and threshold voltage over time are given in Fig. 3(a,b), showing very stable performance under continuous bias stress at *V*_GS_ = *V*_DS_ = −5 V for 25000 seconds. It should be noted that such bias conditions represent the worst case for sensor applications, since for most of cases, the OFET will be biased with smaller voltages and in turn a less current density, as discussed later. Continuous bias stress was also performed for other three devices, which presented similarly good bias stress stabilities in the ambient air as shown in Fig. S3. The fabricated devices without any encapsulation were stored in an uncontrolled environment for four weeks with the recorded humidity and temperature given in Fig. S4(a). The transfer electrical characteristics were measured during the storage, as shown in Fig. 3(c), and the extracted relative changes of mobility and threshold voltage over time are given in Fig. 3(d). The results show stable electrical properties of the device during storage in ambient air. The measurement results for all measured three devices in Fig. S4(b) well prove the reproducibility.

The results prove stable electrial properties of the devices during storage in ambient air. The device stability performance in this work is the best among all reported solution processed or printed OFETs, and is even comparable to previous work with well designed encapsulation^22^. The excellent stabilities are attributed to the air stable and water repellent semiconductor layer and the dielectric materials used in the device. TIPS-pentacene has shown to be an air stable organic semiconductor material with a deep LUMO level of −3.42 eV, and functional groups at the 6 and 13 positions of the molecule protecting the central aromatic ring from oxidation^51^,^52^,^53^. The deposited film from blend of TIPS-pentacene and PS is hydrophobic to be water repellent with a measured water contact angle of 103° as shown in Fig. S5. Blending of small molecule organic semiconductor such as TIPS-pentacene with insulating polymer binder has been reported to be able to improve the crystallization control to obtain a highly crystalline thin film channel^39^,^46^,^54^. As a result, a high quality semiconductor/dielectric interface of low trap states can be formed, which is also important to reduce bias stress induced instabilities.

To prove the importance of the gate dielectric on the device stabilities, devices in the same structure but with PVC and polyvinyl alcohol (PVA) as the gate dielectric layer, respectively, were fabricated on glass substrates for stability comparison, as shown in Fig. S6. It can be seen that both devices present similar low voltage operation properties, indicating the approach for low voltage operation is applicable to different gate dielectric materials. However, since the polar PVA film contains hydroxyl groups (-OH groups) and easily absorbs water, as shown in Fig. S7, the devices present much poorer operational and storage stabilities than the devices with a PVC gate dielectric layer.

The excellent operational and storage stabilities enable using the channel of the OFET as the sensitive layer for long-term reliable vapor or gas sensing without needs of complicated design such as differential sensing architecture^55^,^56^.

### Demonstration of ammonia sensing

**Figure 4:**
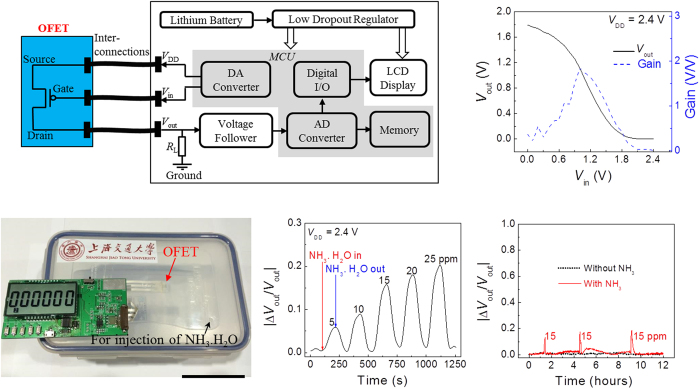
Demonstration of ammonia sensing. (**a**) The circuit schematic of the test system including the OFET sensor tag and the readout circuit board. (**b**) The measured voltage transfer curve between the *V*_out_ and *V*_in_, the bias voltage of *V*_in_ during the NH_3_ test is set as the value to achieve the maximum voltage gain. (**c**) The photograph of the test system. The sensor tag was placed in a plastic container and connected to the test board with a plastic strip. The ammonia water is directly injected into the container via a hole on the top of it. The scale bar is 5 cm. (**d**) The measured relative change of *V*_out_ over time upon NH_3_ exposure of different estimated concentrations from 5 ppm to 25 ppm. (**e**) Long-term sensing performance of the OFET for 12 hours’ continuous test with the system.

NH_3_ sensing in ambient air environment is taken as an example to examine the feasibility of integrating the fabricated low voltage and stable OFET in a low power electronics system for reliable sensing. The measured *I*_D_−*V*_GS_ characteristics in ambient air without NH_3_ exposure, upon NH_3_ exposure and removal of NH_3_ exposure, are compared in Fig. S8. Upon NH_3_ exposure, there is significant change of the *I*_D_−*V*_GS_ curve with a negative *V*_th_ shift of 0.25 V, and a decrease in mobility from 0.6 cm^2^/(V.s) to 0.3 cm^2^/(V.s). NH_3_ has large dipole moment of about 1.46 debye. When NH_3_ molecules are absorbed (mostly at the grain boundaries^57^), the dipolar nature of the NH_3_ molecule produces an electric field capable of strongly binding mobile charges. Then more charges will have to be injected into the active layer in order to turn on the device, which means a negative shift in *V*_th_ for a p-type OFET. Moreover, the dipolar nature of NH_3_ induced charge-dipole interactions is likely to degrade the charge transport in the channel by increasing energetic disorders^58^,^59^, which in turn reduces the field-effect mobility. Upon removal of the analyte, the *I*_D_−*V*_GS_ curve is almost completely restored to its original condition, indicating that the NH_3_ analyte molecules interact with the semiconductor film surface through weak physical absorption (Van del Waals force). After initial studies of its recoverable response to the NH_3_ analyte, the fabricated OFET sensor tag was integrated into a self-designed battery powered readout system to evaluate the long-term sensing performance. The supply voltage (*V*_DD_) of the OFET sensor tag is 2.4 V. The circuit schematic of the whole system including the OFET sensor tag and the readout circuit board is illustrated in Fig. 4(a). The drain current of the OFET is converted to a voltage output signal (*V*_out_) through a load resistor (*R*_L_) in the circuit board, which is then processed by the circuit blocks including a voltage follower and a 12 bit analog-to-digital converter (ADC). The obtained data are stored in the embedded memory in the MCU for collection, and can also be displayed on a liquid crystal display (LCD) screen in real time through a digital I/O interface. The measured voltage transfer curve between *V*_out_ and the bias voltage at the gate of the OFET (*V*_in_) is given in Fig. 4(b). *V*_in_ is set as the value to achieve the maximum voltage gain. The DC power consumption (*P*_DC_) of the OFET sensor tag can be estimated to be around 50 nW (*P*_DC_ ≈ *V*_DD_ × *I*_D_), which is the lowest reported value among all reported OFET based sensors^60^,^61^,^62^,^63^.

To evaluate the performance for long-term NH_3_ sensing in ambient air, the OFET sensor tag was placed in a plastic container and connected to the readout circuit board outside through a plastic conductive strip, as illustrated in Fig. 4(c). Delivery of the analyte into the container is through direct injection of ammonia water (NH_3_.H_2_O) using a micropipette via a hole on top of the container. The concentration of NH_3_ in the container can be roughly estimated according to the volume of the container, the amount of the injected NH_3_.H_2_O and its concentration as described in the Experimental part. The detected concentration should be less than the estimated value, since the NH_3_ molecules might not distribute evenly in the container with the upper region of the container, where the sensor is located, having lower concentration than the bottom part. The sensor tag was taken out of the container for removal of the analyte exposure. A video recording the whole procedure is given in the Supplementary. Fig. 4(d) shows the response of the detected signal over time upon NH_3_ exposure of different estimated concentrations from 5 ppm to 25 ppm. The device shows relatively fast response upon the NH_3_ exposure and removal of the exposure. The response time is also related to the volatilization and diffusion speed of NH_3_ molecules after the ammonia water is injected into the container. Nearly full recovery for all test cycles prove the re-usable feature of the device, which is attributed to weak physical absorption of the analyte to the semiconductor layer surface as mentioned above. Long-term sensing performance was characterized further by continuous test of the OFET for 12 hours with the system. A fixed amount of amonnia water (20 μL) was injected into the container for three times during the period, corresponding to an estimated concentration of 15 ppm. The sensor tag was taken out of the container and then put back after each injection. A same system being running simultaneously without injection of ammonia water was used as the reference. Figure 4(e) shows the test results for the sensor system with repeated NH_3_ exposures, and the reference one being run without exposure to NH_3_. It can be seen that, the OFET sensor is very stable without NH_3_ in the ambient air. The results prove the capability of the device for long-term reliable vapor or gas sensing in ambient air.

## Discussions

Due to material and process constraints, there is a hurdle to achieve both low voltage operation and good stabilities for printable OFETs, especially in an unencapsulated structure. The approach of reducing the sub-gap DOS at the channel is proved to be an effective way to break this deadlock. Based on this idea, a thick low-*k* non-polar polymer dielectric layer, which has a wide range of material choices, can be used to realize low voltage OFETs with better compatibility with printing processes. It is also shown that both storage and operation instability issues can be addressed by using air stable and water repellent semiconductor and dielectric materials. The fabricated devices with IJP Ag electrodes present a steep *S* less than 100 mV/decade and a high ON/OFF current ratio larger than 10^6^, which can help to achieve large detection range for sensing in the subthreshold regime, when the device interfaces with low voltage signal processing silicon chips. The low voltage and also low current density properties of the OFETs results in ultra-low DC power consumption of the sensor tag at the level of around 50 nW during operation, which is the lowest among all reported OFET based sensors, and is attractive for mobile or wearable applications^64^. The demonstration of the OFET being operated in a low voltage battery powered electronic system for long-term and repeatable sensing of the NH_3_ exposure prove the above excellent features of the devices and the capability for practical applications. Moreover, the semiconductor material (blend of TIPS-pentancene and PS), the gate dielectric material (PVC) and the Ag ink used in this work are all commercially available, showing great potential of the technology to be commercially competitive. The work also indicates the importance of engineering the device design with available materials for development of OFET technology.

In conclusion, by reducing the sub-gap DOS at the channel, this work develops fully printable low voltage OFETs with ultra-low power and excellent device stabilities during storage and operation. The first demonstration of an OFET being operated in a battery powered low voltage electronic system for long-term and reliable vapor sensing well prove the device design to be an ideal solution to address the issues with printable OFETs. This work would open a new route to develop a commercially viable printed OFET technology platform for ubiquitous sensor applications.

## Experimental

### Materials and device fabrication

The structure of the fabricated bottom-gate bottom-contact (BGBC) OFETs on flexible substrate is illustrated in Fig. 1(a). A 125-μm-thick polyethylene naphthalate (PEN) foil of 6 cm × 6 cm size was used as the substrate. Poly(vinyl cinnamate) (PVC) solution in chlorobenzene at a concentration of 50 g/L was spin-coated onto the substrate at 3000 r/min, followed by a cross-linking process through UV treatment (UV Curer KW-4AC) for 20 min and then heating at 100 °C for 1 hour, to form the planarization layer. The wavelength of UV radiation was 254 nm. The molecular weight (*M*_W_) of used PVC is 95000. Gate electrodes were printed onto the planarization layer from a type of metal-organic precursor Ag ink (Jet-600C, Hisense Electronics) using a piezoelectric inkjet printer (DMP2831, Dimatix) with a 10 pL cartridge. The cartridge temperature was maintained at 40 °C, while the plate was kept at room temperature. The drop space and print height were set to be 40 μm and 0.8 mm, respectively. The printed Ag gate was sintered at 145 °C for 15 min in the ambient air. Then the same processes as that for the planarization layer were used to deposit the PVC gate dielectric layer. The source/drain electrodes were formed using the same process for the gate electrodes, defining the channel length and width of 80 μm and 1600 μm, respectively. Subsequently, the source/drain electrodes were modified with self-assembled-monolayer (SAM) by immersing the sample in a 5 × 10^−3 ^mol/L solution of perfluorobenzenethiol (PFBT) in ethanol for 2 min and then rinsed with ethanol. The surface of Ag electrodes was treated by PFBT to form good contacts with the organic semiconductor^65^. Before depositing the semiconductor layer, the sample was placed on a glass slide as an inclined support to form a tilted angle of 10°. The semiconducting layer was finally deposited by drop-casting a solution made by mixing 6,13-bis(triisopropylsilylethynyl)-pentacene (TIPS-pentacene) and polystyrene (PS) at 10 mg/ml concentration of solids in chlorobenzene (3:1 ratio by volume), followed by an annealing process at 100 °C for 30 min in ambient air environment. The *M*_W_ of used PS is 524000. All the processes were completed in a non-cleanroom lab environment.

### Material and device characterization

The polarized optical micrograph was taken with a Cakon-XPF-300C microscope. X-ray diffraction (XRD) spectrum of the semiconductor film was characterized with Bruker-AXS D8 Advance system. The surface roughness was measured using a BioScope Veeco atomic force microscope. A WK6515B precision impedance analyzer was used for capacitance measurement. Film thickness was characterized by a KLA-Tencor D-120 Stylus Profiler. The contact angle measurement was carried out with a Solon-200B contact-angle analyser. The electrical properties of the devices were tested with a Keithley 4200 semiconductor characterization system. All measurements were performed at room temperature in ambient air environment.

### Readout circuit board

The circuit schematic of the test system including the OFET sensor tag and the readout circuit board is illustrated in Fig. 4(a). The drain current of the OFET is converted to a voltage output signal (*V*_out_) through a load resistor (*R*_L_) in the circuit board, the *R*_L_ is 50 MΩ. Before processed by MCU, the *V*_out_ is buffered by an operational amplifier Microchip MCP6002, which is connected and used as a voltage follower. The analog-to-digital conversion of *V*_out_ is performed with a 12 bit analog-to-digital converter (ADC) in Texas Instruments MCU MSP430F2618. The digital data *V*_out_ is then stored in the embedded memory of MCU. In parallel, the data is also displayed on a liquid crystal display (LCD) screen in real time through a digital I/O interface. A Holtek HT1621 is used as the LCD driver.

The MCU and LCD are powered by a 3.7 V lithium battery through a low dropout regulator Texas Instruments LP2982. The operation voltage of the MCU and LCD is 3.3 V, which is provided by the low dropout regulator. The supply voltage (*V*_DD_) and bias voltage (*V*_in_) of OFET are provided by the digital-to-analog converter (DAC) of MCU.

Source code written in C programming language is used to control the whole readout circuit board.

### NH_3_ sensing system

The photograph of the NH_3_ test system is shown as Fig. 4(c). A 1.2 L plastic container was used as the test chamber. The OFET sensor tag was cut from the 6 cm × 6 cm flexible sample with a scissor. The tag was placed in the plastic container and connected to the readout circuit board outside through a plastic conductive strip. The plastic conductive strip was fabricated by dispensing three lines of silver paste on polyethylene terephthala (PET), followed by heating at 110 °C for 20 min. The width, length and space between each other of the conductive silver lines were 2 mm, 100 mm and 2 mm, respectively. The OFET sensor tag was attached to one side of the plastic conductive strip with double-sided tape. Then the gate/source/drain electrode of OFET sensor tag was connected to each conductive silver line on the conductive strip by dispensing silver paste, followed by heating at 100 °C for 20 min. The other side of conductive strip was connected with readout circuit board through a Flexible Flat Cable (FFC) socket.

Delivery of the analyte into the container is through direct injection of NH_3_.H_2_O using a micropipette via a hole on top of the container, the hole will be covered with a transparent tape after the injection. During the test, 20 μL of NH_3_.H_2_O with corresponding concentration was injected into the container. The concentration of NH_3_ (*C*_NH3_) in the container can be roughly estimated as:


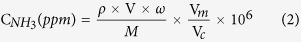


where *ρ* (0.91 g/mL) is the density of NH_3_.H_2_O, *V* (20 μL) is the volume of the injected NH_3_.H_2_O, 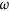 is the mass ratio of the NH_3_ in NH_3_.H_2_O, *M* (17 g/mol) is the molar mass of NH_3_, *V*_m_ (22.4 L/mol) is the molar volume of ideal gas, and *V*_c_ (1.2 L) is the volume of the plastic container. Assuming that NH_3_ distributes evenly in the container after the injection, a 20 μL NH_3_.H_2_O with 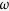 of 0.075% corresponds to a concentration of 15 ppm.

## Additional Information

**How to cite this article**: Feng, L. *et al.* Unencapsulated Air-stable Organic Field Effect Transistor by All Solution Processes for Low Power Vapor Sensing. *Sci. Rep.* **6**, 20671; doi: 10.1038/srep20671 (2016).

## Supplementary Material

Supplementary Information

## Figures and Tables

**Table 1:** Comparisons of ON/OFF ratio, the voltage swing, and the extracted *N*_SS_ at the semiconductor/dielectric interface for our work and other OFETs.

Work	Gate Dielectric	Thickness [nm]	*C*_i_ [nF/cm^2^]	Semiconductor	Source/Drain Electrodes	Substrate	ON/OFF Ratio	Voltage Swing [V]	*N*_SS_ [eV^−1^cm^−2^]
34	P(VDF-TrFE-CFE) (SP)	160	330	PBTTT (SP)	Au (VP)	Glass	10^6^	2.5	1.4 × 10^12^
45	ODPA/AlOx (SP/VP)	2.1/3.6	700	Pentacene (VP)	Au (VP)	Glass	10^6^	2	3.0 × 10^12^
46	Cytop (SP)	900	2.1	TIPS-pentacene (SP)	Au (VP)	Glass	10^6^	60	5.6 × 10^11^
47	D207 (SP)	360	6.9	S1200 (SP)	Ag (SP)	Parylene	10^6^	6	3.9 × 10^11^
48	Teflon (SP)	200	8.4	diF-TES-ADT (SP)	Ag (SP)	PEN	10^6^	25	9.8 × 10^11^
49	PVP (SP)	900	3.8	TIPS-pentacene (SP)	Ag (SP)	Polyarylate	10^5^	60	9.7 × 10^11^
50	ODPA/AlOx (SP/VP)	2.5/4	450	TIPS-Pentacene (SP)	Au (VP)	p^++^ Si Wafer	10^6^	2	1.91 × 10^12^
Our work	PVC (SP)	295	10.2	TIPS-Pentacene (SP)	Ag (SP)	PEN	10^6^	3	3.9 × 10^10^

Note: SP, solution processed; VP, vacuum processed; D207, Merck lisicon D207; S1200, Merck lisicon S1200; diF-TES-ADT, 2,8-difl uoro-5,11-bis(triethylsilylethynyl)anthradithiophene; PEN, polyethylene naphthalate; PVP, poly-4-vinylphenol; TIPS-Pentacene, 6,13(bis-triisopropylsilylethynyl) pentacene; P(VDF-TrFE-CFE), poly(vinylidene fluoride-trifluoroethylene-chlorofluoroethylene) PBTTT, poly(2,5-bis(3-dodecylthiophene-2-yl)thieno[3,2-b]thiophenes); ODPA, n-octadecylphosphonic acid; PVC, poly (vinyl cinnamate).
