# Supplementary material for: Unencapsulated Air-stable Organic Field Effect Transistor by All Solution Processes for Low Power Vapor Sensing
Source: Sci Rep. 2016 Feb 10;6:20671. doi: 10.1038/srep20671 (PMC4748241; doi:10.1038/srep20671)
Supplement: Supplementary Information [file srep20671-s1.pdf]

SUPPLEMENTARY INFORMATION

Unencapsulated Air-stable Organic Field Effect Transistor by All Solution Processes for Low Power Vapor Sensing

Linrun Feng[1 §], Wei Tang[1 §], Jiaqing Zhao[1], Ruozhang Yang[1], Wei Hu[1], Qiaofeng Li[1], Ruolin Wang[1] & Xiaojun Guo[1*]

[1] National Engineering Laboratory of TFT-LCD Materials and Technologies, Department of Electronic Engineering, Shanghai Jiao Tong University, Shanghai 200240, China. [§]These authors contributed equally to this work. Correspondence and requests for materials should be addressed to X.G. (e-mail: x.guo@sjtu.edu.cn).

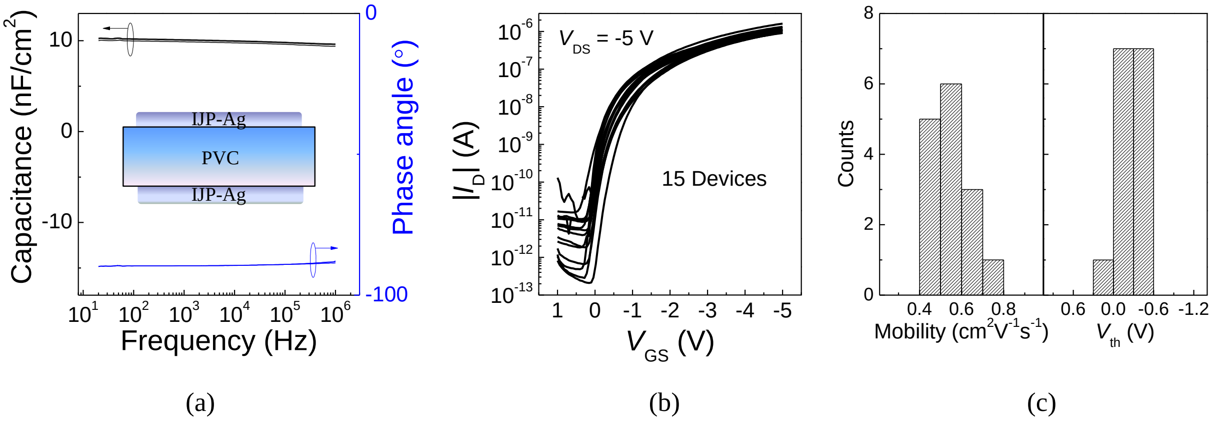

Supplementary Figure S1 | Measured dielectric capacitance and device uniformity. (a) The measured capacitance as a function of frequency for PVC dielectric for three devices based on an IJP-Ag/PVC/IJP-Ag test structure, showing identical results. The dielectric constant of PVC is calculated to be 3.4 at 20 Hz. (b) The measured transfer characteristics of 15 devices over the 6 cm×6 cm size substrate. (c) The histograms of extracted mobility and threshold voltage (V_{th}) for the 15 devices.

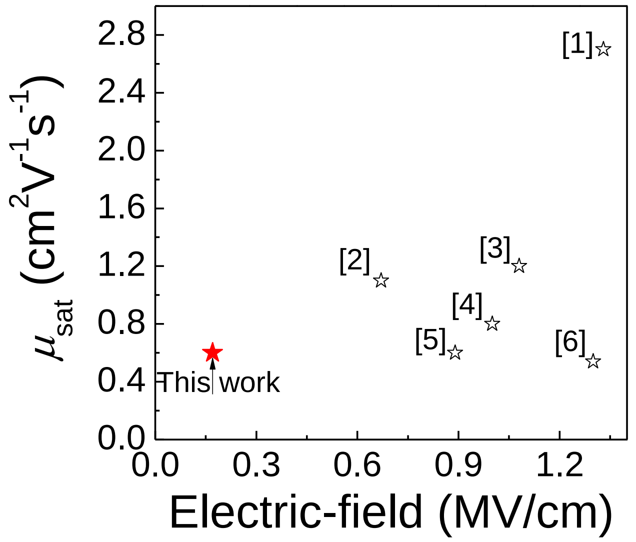

Supplementary Figure S2 | Comparison of the extracted saturated mobility and the gate electric field values for the reported TIPS-pentacene based OFETs. The comparison indicated ordinary mobility of TIPS-pentacene reported here with a low gate electric field.

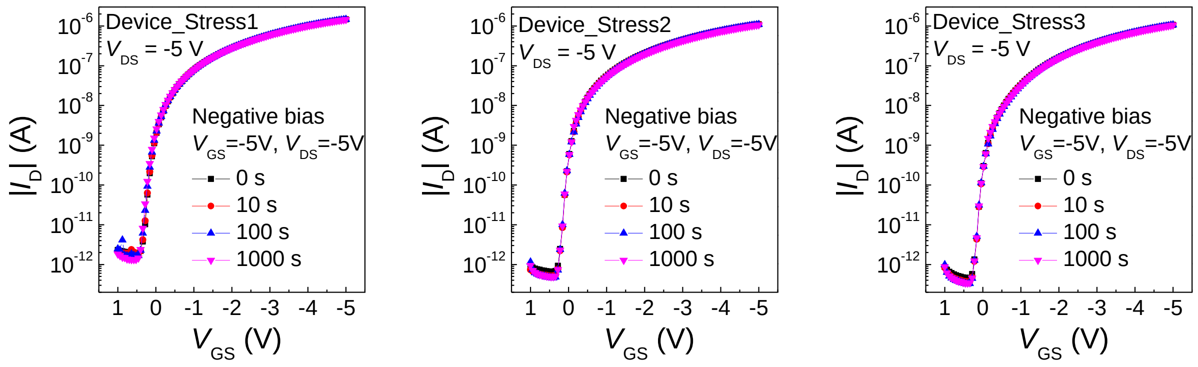

Supplementary Figure S3 | The measured operational stabilities of three OFET devices. The transfer characteristics were measured under continuous bias stress for 1000 s with an applied bias voltage of V_{GS} = V_{DS} = -5V.

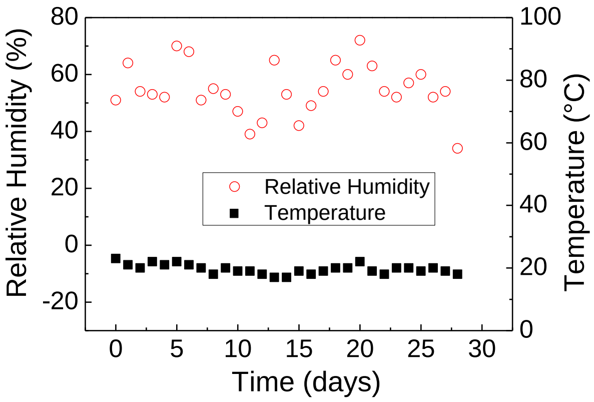

(a)

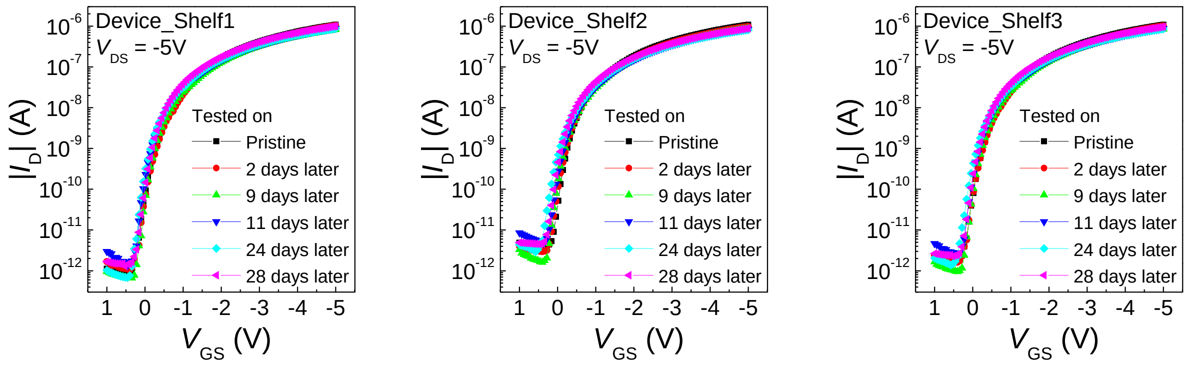

(b)

Supplementary Figure S4 | Measurment of electrical properties of three OFET devices during storage in ambient air. (a) The recorded relative humidity and temperature during the four week for the measurement. (b) The measured transfer characteristics of three OFET devices during storage in ambient air environment for four weeks.

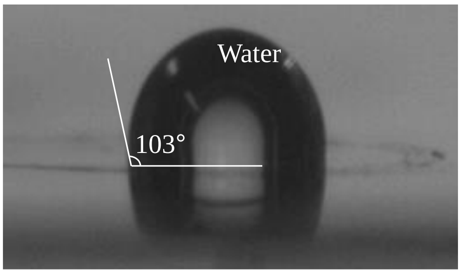

Supplementary Figure S5 | Measured water contact angle. The measured water contact angle of TIPS-pentacene/PS film, indicating the blend semiconductor is hydrophobic.

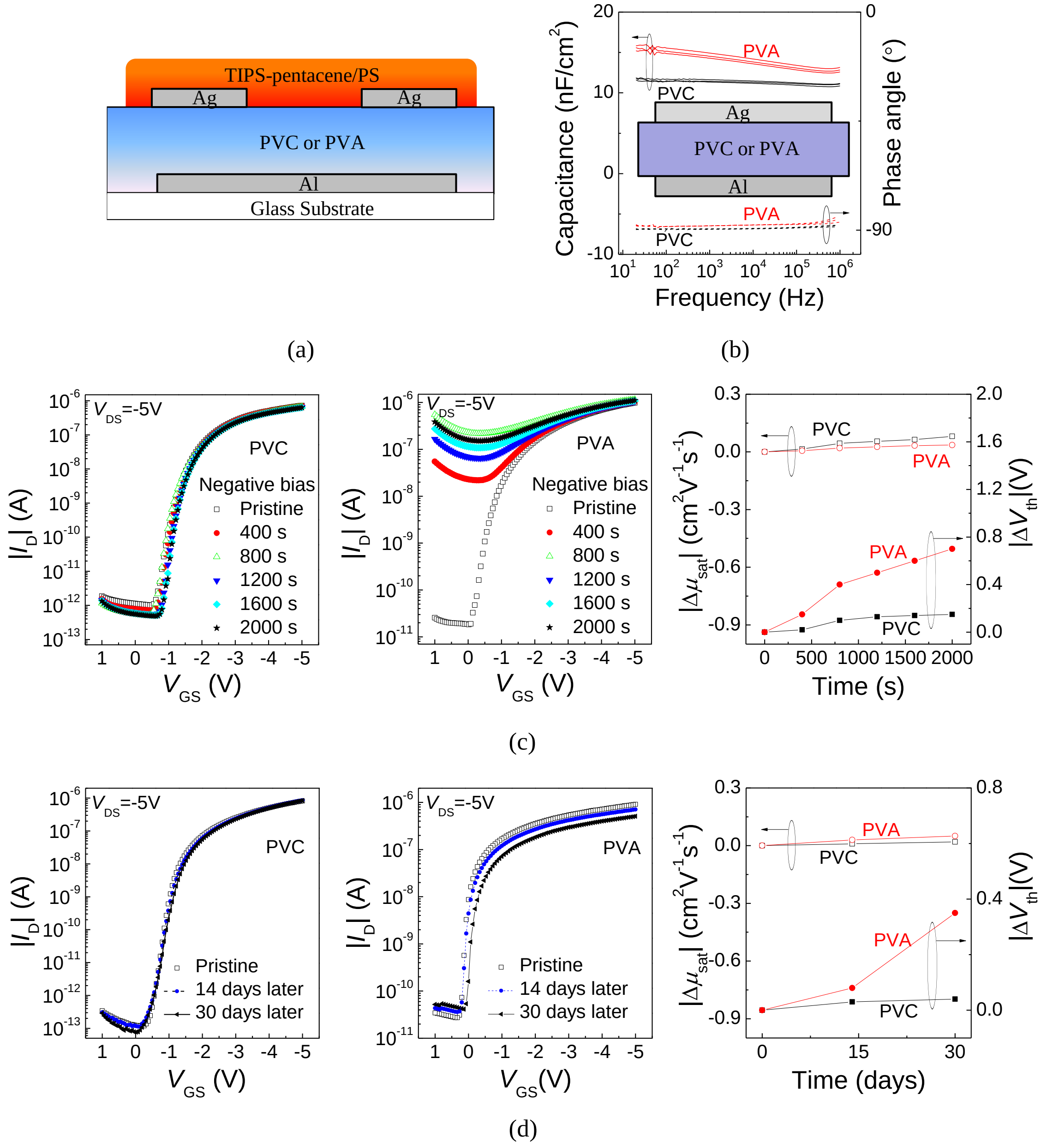

Supplementary Figure S6 | Comparison of the stabilities of the devices with PVC and PVA gate dielectric layers. (a) The schematic diagram of the fabricated devices with PVC or PVA dielectric. (b) The measured capacitance as a function of frequency for both PVC and PVA dielectrics based on an Al/PVC (or PVA)/Ag test structure (three devices were measured for each condition). At the frequency of 20 Hz, the dielectric constant of PVA and PVC were calculated to be 6.7 and 3.4, respectively. (c) The measured bias stress stability of the devices with an applied bias voltage of V_{GS} = -5 V, V_{DS} = -0.5 V for 2000 s, with the plotted relative changes of extracted mobility and threshold voltage of the devices as a function of bias time. (d) The measured transfer characteristics of the devices during storage, with the plotted relative changes of extracted mobility and threshold voltage of the devices as a function of storage time.

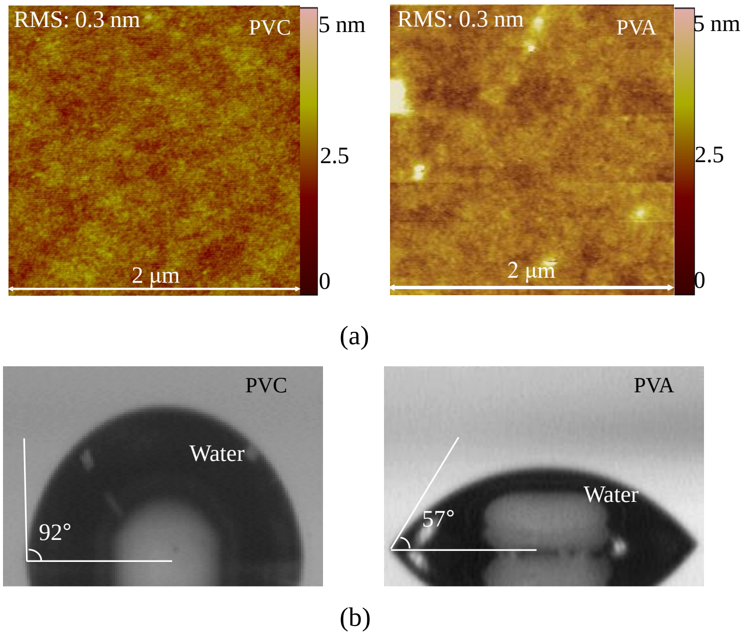

Supplementary Figure S7 | Characterization of the PVC and PVA dielectrics. (a) The measured surface roughness with atomic force microscope (AFM) for the PVC and PVA dielectric. Both the dielectrics present a smooth pin-hole free surface with a root-mean-square (RMS) roughness of about 0.3 nm, which is prerequisite to form a low trap-state dielectric/semiconductor interface. (b) The measured water contact angle for the PVC and PVA dielectric. PVC is much more hydrophobic with a contact angle against water of 92° compared with 57° of PVA's.

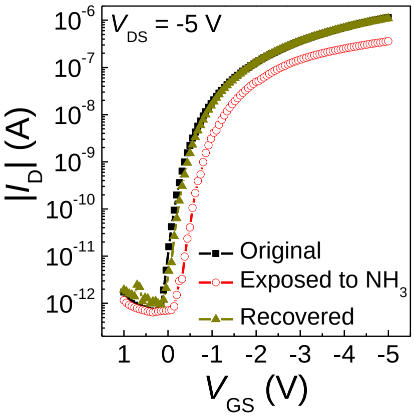

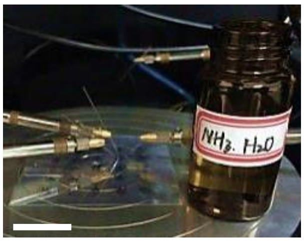

	V_{th} (V)	Mobility ($cm^2V^{-1}s^{-1}$)
Original	-0.5	0.6
Exposure	-0.75	0.3
Recovery	-0.5	0.6

(a) (b)

Supplementary Figure S8 | The measured NH_3 response. (a) The measured transfer characteristics of the device in ambient air without NH_3 exposure, upon NH_3 exposure and removal of NH_3 exposure. (b) The photograph of the test during the NH_3 exposure. The scale bar is 2 cm. The NH_3 exposure was realized by opening a bottle of high concentration NH_3.H_20 with a mass ratio of 25% close to the device during test. The removal of NH_3 exposure was carried out by closing the bottle and moving the bottle away from the device. The extracted V_{th} and mobility values for the transfer characteristics of (a) is shown in the table.

References

1. Giri, G. *et al*. High-mobility, aligned crystalline domains of TIPS-pentacene with metastable polymorphs through lateral confinement of crystal growth. *Adv. Mater*. **26**, 487-493 (2014).
2. Hamilton, R. *et al*. High-performance polymer-small molecule blend organic transistors. *Adv. Mater*. **21**, 1166-1171 (2009).
3. Park, S. *et al*. High mobility solution processed 6,13-bis (triisopropyl-silylethynyl) pentacene orgnic thin film transistors. *Appl. Phys. Lett*. **91**, 063514 (2007).
4. Kjellander, B.*et al*. Inkjet printing of TIPS-PEN on soluble polymer insulating films: a route to high-performance thin-film transistors. *Adv. Mater*. **22**, 4612-4616 (2010).
5. Hwang, K. *et al*. Top-gate organic field-effct transistors with high environmental and operational stability. *Adv. Mater*. **23**, 1293-1298 (2011).
6. Kang, J. *et al*. Structure and properties of small molecule-polymer blend semiconductors for organic thin film transistors. *J. Am. Chem. Soc.* **130**, 12273-12275 (2008).
